# The Correct Principles of Treatment for Angular Curvature of the Spine

**Published:** 1872-09

**Authors:** 


*The Correct Principles of Angular Curvature of the Spine.* By BENJAMIN LEE, A. M., M. D. Philadelphia: J. B. Lippincott & Co., 1872.

The labors of such men as Drs. Davis, and Taylor and Lee have done much to rescue the treatment of Spinal Curvature from that system of barbarity with which it was formerly treated. The setons, issues, moxas, &c., with which diseases of the spinal column were treated; together with the species of support (?) which it was made to undergo may now be viewed with a curiosity and horror almost equal to that with which we would gaze upon the wheel and rack of the days of the inquisition.

The application of the mind of the scientific physician to the department of mechanical support to the spine has produced such results that we can unhesitatingly say with the author: "first, that an *early recognition* of the disease will in a great number of cases enable us to arrest it *before deformity has been produced*; secondly, that *mechanical treatment* is of vastly more importance than medication."

This little work is divided into two parts—Antero-Posterior Support, and Modified Suspension.

We are glad to see that the author repudiates the idea once so generally accepted as to the tuburcular nature of diseases of the spine. The clinical and post mortem observations constantly being made are directly contradictory to this theory, and we hail with pleasure the time when it will cease to occupy the minds of medical practitioners.

The cases related in this work are plainly stated and the observations clearly drawn.

We have been highly pleased in its perusal, and would recommend it to any of our friends seeking information concerning Angular Curvature of the Spine.

